# From the patient to the population: Use of genomics for population screening

**DOI:** 10.3389/fgene.2022.893832

**Published:** 2022-10-24

**Authors:** Chloe Mighton, Salma Shickh, Vernie Aguda, Suvetha Krishnapillai, Ella Adi-Wauran, Yvonne Bombard

**Affiliations:** ^1^ Genomics Health Services Research Program, St. Michael’s Hospital, Unity Health Toronto, Toronto, ON, Canada; ^2^ Institute of Health Policy, Management and Evaluation, University of Toronto, Toronto, ON, Canada; ^3^ Centre for Medical Education, School of Medicine, Cardiff University, Cardiff, United Kingdom

**Keywords:** population screening, tier 1 conditions, hereditary breast and ovarian cancer (HBOC), lynch syndrome, familial hypercholestelemia, genetic testing

## Abstract

Genomic medicine is expanding from a focus on diagnosis at the patient level to prevention at the population level given the ongoing under-ascertainment of high-risk and actionable genetic conditions using current strategies, particularly hereditary breast and ovarian cancer (HBOC), Lynch Syndrome (LS) and familial hypercholesterolemia (FH). The availability of large-scale next-generation sequencing strategies and preventive options for these conditions makes it increasingly feasible to screen pre-symptomatic individuals through public health-based approaches, rather than restricting testing to high-risk groups. This raises anew, and with urgency, questions about the limits of screening as well as the moral authority and capacity to screen for genetic conditions at a population level. We aimed to answer some of these critical questions by using the WHO Wilson and Jungner criteria to guide a synthesis of current evidence on population genomic screening for HBOC, LS, and FH.

## Introduction

Genomic medicine is expanding from a focus on diagnosis at the patient level to prevention at the population level. As test costs fall, it could become feasible to screen pre-symptomatic individuals through public health-based approaches, rather than restricting testing to high-risk groups. Indeed, pilot initiatives in which hundreds of thousands of individuals will undergo genomic screening are being launched in health systems in the United States (U.S.) (Carey et al., 2016; Schwartz et al., 2018; Lacaze et al., 2019; Grzymski et al., 2020), the United Kingdom (U.K.) (Genomics England, 2021), and Australia (Rowley et al., 2019; Lacaze et al., 2022). Leading hereditary conditions for consideration in population screening include hereditary breast and ovarian cancer syndrome (HBOC), Lynch syndrome (LS), and familial hypercholesterolemia (FH). These conditions are prioritized for screening due to their under-ascertainment using current screening approaches and the availability of evidence-based interventions to reduce morbidity and mortality (Centers for Disease Control and Prevention OoPHG, 2022).

Traditional methods to identify cases with HBOC, LS, and FH include genetic testing for patients meeting clinical, ethnicity or family-history based criteria (Hampel et al., 2008; Schofield et al., 2014; Klančar et al., 2015; Tognetto et al., 2017; Groselj et al., 2018; Gupta et al., 2019; Daly et al., 2020; Kunnackal John et al., 2021; Zuurbier et al., 2021). However, these targeted approaches have been found to miss a substantial proportion of individuals who harbor pathogenic variants. For example, >50% of individuals with pathogenic *BRCA1* and *BRCA2* (*BRCA1/2*) variants are missed by family history-based criteria (Metcalfe et al., 2010a; Gabai-Kapara et al., 2014; Manchanda et al., 2015a). The availability of large-scale next-generation sequencing (NGS) strategies and preventive options for these conditions makes it increasingly feasible to screen pre-symptomatic individuals through public health-based approaches, rather than restricting testing to high-risk groups.

This raises anew, and with urgency, questions about the limits of screening as well as the capacity to screen for genetic conditions at a population level, or in other words, population genomic screening. We use the term “population genomic screening” to refer to germline DNA testing among an unselected, asymptomatic population with the aim of identifying individuals with pathogenic/likely pathogenic (henceforth, “pathogenic”) variants. Key issues to scaling up population genomic screening include the optimal testing approach, penetrance of these conditions in the general population, clinical effectiveness, cost-effectiveness, acceptability, health system capacity to implement such a program, ethical issues such as overdiagnosis, access challenges and equity.

Decisions about screening are expected to align with the World Health Organization principles of screening. These criteria, developed by Wilson and Jungner in 1968, inform decision-making around disease screening and generally include considerations of the nature of the disease, test characteristics, and the availability, effectiveness and acceptability of preventive interventions or treatments (Table 1) (Wilson and Jungner, 1968). Since its publication, Wilson and Jungner’s criteria have been widely accepted, modified and used by decision-makers across the world to guide screening decisions. Whereas the Wilson and Jungner criteria were developed for programs aiming to enable early detection and intervention for individuals with early stages of a disease, population genomic screening programs would identify those with a genetic predisposition to disease. The identification of a pathogenic variant in an asymptomatic individual through genetic screening is not equivalent to a clinical diagnosis of the associated disease (Murray, 2016; Murray et al., 2021). Given the complexity of policy decision-making for genetic tests and genetic screening programs, various frameworks and sets of decision criteria have been developed to guide these decisions (Sanderson et al., 2005; Burke and Zimmern, 2007; Andermann et al., 2008; Teutsch et al., 2009; Andermann et al., 2011; National Academies of Sciences Engineering and Medicine, 2017; Pitini et al., 2019). While these newer frameworks and decision criteria share core elements with Wilson and Jungner such as those related to the natural history of the condition, the effectiveness of the test, and effectiveness of preventive interventions, newer frameworks extend Wilson and Jungner’s criteria to include considerations related to implementation issues such as health service delivery, ethics, and equity. However, these more recent criteria for genomic evaluation have not been universally adopted, and different health systems vary in which criteria are used in policy decisions, if any. Given the lack of a universally accepted set of decision criteria for genomic screening, and the continued relevance of the fundamental principles of Wilson and Jungner, we will use the Wilson and Jungner criteria to guide a synthesis of the current evidence on population genomic screening for leading gene-condition pairs. In addition, we also discuss ethical and equity considerations. While these are absent from the original Wilson and Jungner criteria, they are increasingly important in decision frameworks for genomic screening programs (Andermann et al., 2008; Pitini et al., 2019) and are commonly considered across various frameworks and sets of decision criteria for genomic technologies (Burke and Zimmern, 2007; Andermann et al., 2008; Teutsch et al., 2009; Andermann et al., 2010; Botkin et al., 2010; Andermann et al., 2011). We highlight policy and practice issues as well as future research priorities to inform the design of population genomic screening programs to maximize population benefits and minimize harms.

**TABLE 1:** Wilson and Jungner’s principles for disease screening (Wilson and Jungner, 1968).

#	Principle
1	The condition sought should be an important health problem
2	There should be an accepted treatment for patients with recognized disease
3	Facilities for diagnosis and treatment should be available
4	There should be a recognizable latent or early symptomatic stage
5	There should be a suitable test or examination
6	The test should be acceptable to the population
7	The natural history of the condition, including development from latent to declared disease, should be adequately understood
8	There should be an agreed policy on whom to treat as patients
9	The cost of case-finding (including diagnosis and treatment of patients diagnosed) should be economically balanced in relation to possible expenditure on medical care as a whole
10	Case-finding should be a continuing process and not a “once and for all” project

## Is the condition sought an important health problem?

HBOC, LS and FH are characterized by their high penetrance, evidence-based interventions for prevention/treatment and subsequent benefits from the early detection, in line with fundamental principles of screening. The CDC Office of Public Health Genomics (OPHG) designates screening for HBOC, LS, and FH as Tier 1 genomic applications (Centers for Disease Control and Prevention OoPHG, 2022). Tier 1 genomic applications are those which could have a substantial, positive impact on public health based on: 1) A high prevalence of 1 in 200 for HBOC, 1 in 340 for LS and in 1 in 250 for FH in the general populations (exact frequency may vary in certain populations); 2) the under-ascertainment of current strategies; and, 3) established risk-reducing interventions that reduce morbidity and mortality (Abul-Husn et al., 2016; Akioyamen et al., 2017; Manickam et al., 2018; Grzymski et al., 2020; Manickam et al., 2021).

## Is the natural history of the condition adequately understood?

The natural histories of HBOC, LS, and FH are relatively well understood. HBOC is caused by pathogenic variants in *BRCA1/2* which confer substantially elevated risks for female breast cancer, ovarian cancer, and male breast cancer (in particular for *BRCA2* carriers), in addition to increased risks for pancreatic cancer, prostate cancer, and melanoma (The Breast Cancer Linkage Consortium, 1999; Brose et al., 2002; Levine et al., 2003; Lindor et al., 2008; Lynch et al., 2009; Moran et al., 2012; Mavaddat et al., 2013; McKay et al., 2016). While pathogenic variants in other genes including *PALB2, RAD51C, RAD51D*, and *BRIP1* also cause hereditary breast and ovarian cancer, we focus this review on *BRCA1/2* because of the higher frequency of pathogenic variants in the population in these genes, and established clinical management guidelines (Manickam et al., 2018; National Comprehensive Cancer Network (NCCN), 2021a). LS is caused by pathogenic variants in mismatch repair (MMR) genes *MLH1, MSH2, MSH6*, and *PMS2*, as well as deletions in *EPCAM* which lead to silencing of *MSH2.* Affected individuals are at increased risk for colorectal cancer (CRC), endometrial cancer, ovarian cancer, and other cancers (Lindor et al., 2008; Senter et al., 2008; Baglietto et al., 2010; Bonadona et al., 2011; Giardiello et al., 2014). FH, caused by pathogenic variants in *LDLR, PCSK9,* and *APOB,* is characterized by elevated plasma low-density lipoprotein cholesterol (LDL-C) levels, which leads to risks for cardiovascular disease and premature mortality (Youngblom et al., 2016).

Two key issues that inform natural history are penetrance and age of onset. HBOC, LS and FH exhibit high but incomplete penetrance. Although the penetrance (the chance that an individual with the condition will manifest particular features) of the causative genes has been estimated in cohorts ascertained with strong personal and family history of disease, it has yet to be well-established in the general population (Murray et al., 2021). Some studies suggest penetrance in the general population may vary from estimates from family-based studies (Forrest et al., 2022). However, the risk to those identified through population screening will likely still be high enough to warrant clinical intervention, at least in *BRCA*1/2 carriers where there is substantial evidence demonstrating high penetrance even among unselected cases (Chatterjee et al., 2001; Chatterjee and Wacholder, 2001; Antoniou et al., 2005; Chatterjee et al., 2006; Kuchenbaecker et al., 2017; Chen et al., 2020). These studies highlight the importance of evaluating the appropriateness of population genomic screening and subsequent interventions, given the potential for overdiagnosis and overtreatment (to be discussed in a subsequent section, *Ethical considerations*). Adding another layer of complexity to risk prediction, other genetic factors, such as polygenic background, and non-genetic risk factors (e.g., diet, environmental exposures, and clinical risk factors) can also influence the penetrance of these conditions (Fahed et al., 2020).

Based on the age of onset and availability of age-appropriate preventive interventions, the optimal age to initiate screening will vary across target conditions. For example, surveillance and risk-reducing surgeries for HBOC and LS are recommended in adulthood (National Comprehensive Cancer Network (NCCN), 2021a; National Comprehensive Cancer Network (NCCN), 2021b), while pharmacologic treatment of FH can begin in childhood (Gidding et al., 2015). The health outcomes and costs of population screening programs will likely vary depending on the age at which screening and intervention is initiated. Specific considerations related to the target population for each condition are provided throughout the subsequent sections.

## Is there a suitable test or examination?

One element of test performance is validity, which encompasses both “analytic validity” (accuracy in detecting the target genetic variant) and “clinical validity” (accuracy in identifying patients with the target condition) (Bombard et al., 2013). Test selection for population genomic screening should consider what type of genetic variation primarily causes the target condition, and testing laboratories should be equipped to manage gene-specific technical challenges [e.g., *PMS2* pseudogenes (Hegde et al., 2014; Li et al., 2015; Lee et al., 2021a)]. Several laboratory considerations for population genomic screening include whether to perform full gene sequencing or targeted variant testing, whether to test for only known pathogenic variants or also novel variants, and whether to perform deletion/duplication analysis in addition to sequence analysis; each of these decisions will impact test costs and post-test residual risk (Lu et al., 2019). NGS has very high analytic sensitivity and specificity for detecting single-nucleotide variants and small insertions/deletions (Baudhuin et al., 2015; Judkins et al., 2015; Toland et al., 2018), and could be coupled with gene-targeted deletion/duplication analysis to increase detection of disease-causing variants for HBOC, LS and FH (Petrucelli et al., 1998; Idos et al., 2004; Ison et al., 2014). Deletion/duplication analysis is necessary to identify disease-causing variants in *EPCAM*. The use of array-based genotyping in population genomic screening has been found to result in false positives and false negatives compared to NGS or Sanger sequencing (Blout Zawatsky et al., 2021; Bowling et al., 2021). For HBOC, in the Ashkenazi Jewish (AJ) population, there are three founder variants (*BRCA1* c.68_69delAG, *BRCA1* c.5266dupC and *BRCA2* c.5946delT) which are prevalent in ∼2.5% (Roa et al., 1996) of the population. While these variants do account for the majority of pathogenic *BRCA1/2* variants in the AJ population (Walsh et al., 2017), some *BRCA1/2* carriers would be missed if targeted founder variant testing as opposed to NGS was used in population genomic screening among the AJ population (Rosenthal et al., 2015; Solano et al., 2018).

Another aspect of genetic test performance is variant interpretation (Richards et al., 2015). Key issues related to variant interpretation include variants of uncertain significance (VUS) (Burke et al., 192022), discordant variant interpretations between diagnostic laboratories (Garber et al., 2016; Harrison et al., 2017; Iacocca et al., 2018; Lebo et al., 2018; Amendola et al., 2020; Mighton et al., 2021a), variant reclassification over time (Macklin et al., 2018; Mersch et al., 2018; Slavin et al., 2018; Turner et al., 2018; Esterling et al., 2020; Chiang et al., 2021) and recontacting patients with updated results (e.g., changes from VUS to likely pathogenic or pathogenic which may warrant medical follow-up) (Otten et al., 2015; El Mecky et al., 2019). While these issues exist in standard clinical genetic testing, they will be magnified if genomic screening is conducted at a population scale, and will need to be considered in program design/implementation.

A further aspect of test performance is the positive predictive value (PPV), the probability that a patient with a positive result (a reported pathogenic or likely pathogenic variant) has the associated condition (Hagenkord et al., 2020). PPV depends on test characteristics (specificity, sensitivity) and condition prevalence (Akobeng, 2007; Oleske, 2010; Hagenkord et al., 2020). As HBOC, FH, and LS have a lower prevalence in the general population compared to populations ascertained based on family history, this would reduce the PPV of a positive result obtained from population genomic screening compared to a positive result from genomic testing among high-risk populations (Hagenkord et al., 2020). Estimates of PPV for Tier 1 conditions range from 80% to 91%, assuming 99.95% specificity and that one-third of the overall positive rate is likely pathogenic variants and two-thirds are pathogenic variants (Hagenkord et al., 2020). Increasing test specificity can increase the PPV, which laboratories could accomplish by adjusting the reporting cut-off between a positive and a negative result (Lu et al., 2019; Hagenkord et al., 2020). For example, reporting only high confidence likely pathogenic variants can increase specificity (Hagenkord et al., 2020).

## Is there a recognizable latent or early symptomatic stage?

Among these three conditions, there is a pre-symptomatic state that is identifiable by molecular testing for pathogenic variants in the relevant genes (Youngblom et al., 2016; Petrucelli et al., 2022). Therefore, population genomic screening for HBOC, LS, and FH can be used to identify individuals with pathogenic variants in the causative genes who would not otherwise be identified through routine clinical care and could gain benefits from early intervention (Grzymski et al., 2020). Multiple studies have found that population genomic screening identifies carriers of pathogenic variants for HBOC, LS, and FH who were previously unaware of their variant (Buchanan et al., 2020; Grzymski et al., 2020; Abul-Husn et al., 2021; Lee et al., 2021b; Blout Zawatsky et al., 2021).

### Hereditary breast and ovarian cancer

Population genomic screening methods have been found to identify a higher proportion of *BRCA1*/2 carriers than family-history and clinical criteria-based methods (Manchanda et al., 2015a; Manickam et al., 2018; Abul-Husn et al., 2019). In addition to their improved detection rate, *BRCA1*/2 screening programs suggest that penetrance of cancer in families of Ashkenazi Jewish ancestry identified through population screening programs is just as high as in families identified through traditional family history based or clinical criteria methods (Gabai-Kapara et al., 2014).

### Lynch syndrome

Compared to traditional approaches for clinically ascertaining LS cases (e.g., tumor testing followed by germline testing among affected patients or family history-based approaches for unaffected cases (Hampel et al., 2008; Batte et al., 2014; Tognetto et al., 2017; Kahn et al., 2019), a potential benefit of population genomic screening is the identification of a greater number of pre-symptomatic patients which could allow for cancer prevention through enhanced surveillance, chemoprevention with aspirin, and surgical prevention with hysterectomy and bilateral salpingo-oophorectomy. Several studies have found that population genomic screening identified pre-symptomatic individuals with pathogenic variants in LS genes who were unaware of their variant and would be missed by standard approaches to case identification (Buchanan et al., 2020; Grzymski et al., 2020; Abul-Husn et al., 2021; Lee et al., 2021b; Blout Zawatsky et al., 2021).

### Familial hypercholesterolemia

Evidence from clinical testing programs and population-based studies suggest that population genomic screening for FH will lead to benefits. These include increased case detection and short-term improvements, especially when conducted during the pediatric period, given the potential for early intervention through dietary cholesterol reduction, medication, and screening intensity (Smith et al., 2016). Systematic reviews and observational studies have found that universal lipid screening for FH among children and adolescents followed by targeted genetic testing, and cascade testing of relatives, are effective methods for identifying FH cases (Lozano et al., 2016a; Wald et al., 2016; Groselj et al., 2018; Lee et al., 2019; Matsunaga et al., 2021; Zuurbier et al., 2021). The availability and lower costs of lipid screening approaches raises questions about the necessity of using genomic screening as a first tier test to identify FH cases.

## Are there accepted options for surveillance and prevention for high-risk populations?

There are various surveillance and prevention options endorsed by clinical practice guidelines to guide the management of individuals with HBOC, LS and FH.

### Hereditary breast and ovarian cancer syndrome

Although there are guidelines for the management of patients with pathogenic variants in various HBOC genes (National Comprehensive Cancer Network (NCCN), 2021a; Tischkowitz et al., 2021; Manchanda et al., 2022), we are focusing on the Tier 1 genes, *BRCA1* and *BRCA2*. In terms of prevention, bilateral prophylactic mastectomy and risk-reducing salpingo-oophorectomy are highly effective in preventing breast cancer and ovarian/fallopian tube cancers respectively in addition to reducing mortality, though a small residual risk for primary peritoneal cancer remains (National Comprehensive Cancer Network (NCCN), 2021a; Li et al., 2016; Honold and Camus, 2018; Finch et al., 2014).

Among females who decline or defer surgery, early detection options for female carriers of a disease-causing *BRCA1/2* variant usually comprise of a combination of routine mammograms and breast MRIs for breast cancer risks, which are effective at detecting breast cancer among *BRCA1/2*-positive females. MRI is more sensitive than mammography in high-risk females (National Comprehensive Cancer Network (NCCN), 2021a; Warner et al., 2004; Kriege et al., 2004; Leach et al., 2005; Kuhl et al., 2005; Riedl et al., 2007; Sardanelli et al., 2007; Lowry et al., 2012; Lehman et al., 2016). Among high-risk females, MRI in combination with mammography has been found to be more sensitive than either modality alone (Warner et al., 2008; Mann et al., 2019) and to improve overall survival relative to mammography alone (Bae et al., 2020). In an observational cohort study of MRI in combination with mammography among unaffected female *BRCA1/2* heterozygotes, the probability of dying of breast cancer within 20 years was 2% (Warner et al., 2020). For ovarian cancer risks, guidelines from the National Comprehensive Cancer Network (NCCN) suggest that transvaginal ultrasound and CA-125 may be offered at the clinician’s discretion to *BRCA1/2* carriers who have not elected for risk-reducing salpingo-oophorectomy (National Comprehensive Cancer Network (NCCN), 2021a). However, these interventions are of uncertain benefit (National Comprehensive Cancer Network (NCCN), 2021a; Jacobs et al., 2016; Menon et al., 2009) and ovarian cancer screening with transvaginal ultrasound and CA-125 has not been demonstrated to reduce mortality (Menon et al., 2021).

Chemopreventive options are routinely offered in clinical practice given the evidence that they reduce breast cancer risk for all at-risk populations, including *BRCA1/2* carriers (National Comprehensive Cancer Network (NCCN), 2021a; Gronwald et al., 2006; Narod et al., 2000).

For male carriers of *BRCA1*/2 pathogenic variants, recommendations consist of yearly screening with a digital rectal exam and prostate-specific antigen (PSA) blood test initiated by age 40–45 however limited data exists to support the effectiveness of additional screening (breast cancer) (National Comprehensive Cancer Network (NCCN), 2021a; Gao et al., 2019).

Studies with female AJ *BRCA1*/2 carriers identified through population screening indicate acceptability for and high uptake of risk-reducing strategies (Metcalfe et al., 2012; Lieberman et al., 2017). Long-term follow up supports improvements in psychological outcomes such as anxiety (Metcalfe et al., 2012; Manchanda et al., 2015a; Manchanda et al., 2020a; Morgan et al., 2021). In the general population, there is less evidence on the uptake of preventive strategies or outcomes. Several studies indicate that many *BRCA1*/2 carriers identified through population screening do undergo risk-reducing procedures such as surveillance or prophylactic surgery (Buchanan et al., 2020; Lee et al., 2021b; Elhanan et al., 2022). In some cases, HBOC-associated cancers were diagnosed because of the screening initiated based on the genomic screening results (Buchanan et al., 2020).

### Lynch syndrome

For Lynch syndrome, there are strategies for early detection or prevention of CRC and gynaecological cancers. Early detection strategies in LS include recommendations for colonoscopy, endoscopy, and total body examinations (National Comprehensive Cancer Network (NCCN), 2021b; Stjepanovic et al., 2019). Surveillance colonoscopy is effective at reducing CRC burden and improving survival among LS patients (Dove-Edwin et al., 2002; Järvinen et al., 2009; Ladabaum et al., 2015; Stjepanovic et al., 2019), though the optimal intervals for surveillance and age to initiate screening are still areas of investigation (National Comprehensive Cancer Network (NCCN), 2021b; Stjepanovic et al., 2019; Järvinen et al., 2009; Dove-Edwin et al., 2002; Jenkins et al., 2015), especially among patients with *PMS2* variants which may have lower penetrance (National Comprehensive Cancer Network (NCCN), 2021b; Lindor et al., 2006). There is observational evidence that prophylactic hysterectomy and/or bilateral salpingo-oophorectomy effectively reduce the incidence of gynaecological cancers among females with LS (Schmeler et al., 2006) and is routinely recommended for at-risk females (Crosbie et al., 2019); however, evidence on mortality is lacking. Chemoprevention with aspirin is also an option for LS risk management as there is evidence that aspirin reduces risk for CRC and other LS-associated cancers (Burn et al., 2011; Ait Ouakrim et al., 2015), however there is no evidence on the effect of aspirin on mortality (Rubenstein et al., 2015). Endometrial cancer screening has not been proven to benefit LS patients (National Comprehensive Cancer Network (NCCN), 2021b). However, it may be considered at the discretion of the clinician every 1–2 years in conjunction with endometrial biopsy, which is considered a sensitive and specific diagnostic test (National Comprehensive Cancer Network (NCCN), 2021b). Transvaginal ultrasound can be considered among postmenopausal females (National Comprehensive Cancer Network (NCCN), 2021b).

Evidence on the outcomes of population genomic screening for LS beyond detection rate is limited. Several studies of population genomic screening for LS have found that a proportion of individuals with pathogenic LS variants underwent risk-reducing procedures, including colonoscopy and prophylactic surgery (Buchanan et al., 2020; Lee et al., 2021b; Elhanan et al., 2022). Several individuals were diagnosed with LS-associated cancers because of follow-up initiated based on their genomic screening results (Buchanan et al., 2020). However, there is some literature that suggests the uptake of risk-reducing strategies is very low (< 10%) when patients are responsible for communicating their results to their clinicians (Elhanan et al., 2022).

### Familial hypercholesterolemia

Management of heterozygous FH is aimed at primary prevention of atherosclerotic cardiovascular disease through lipid lowering pharmacological therapy, using statins, ezetimibe or PCSK9 inhibitors or other LDL lowering medications, with guidelines recommending initiation at ages 8–10 or earlier based on severity (Carroll et al., 2008; Gidding et al., 2015; Defesche et al., 2017; Kim et al., 2021). Trials have yet to directly compare cardiovascular disease outcomes associated with different pharmacologic treatments for heterozygous FH, and treatment recommendations therefore are based on surrogate outcomes including LDL cholesterol lowering and arterial imaging (Defesche et al., 2017). For example, a systematic review found that statins were effective at lowering LDL-C and total cholesterol (TC) concentration, but there was no evidence on the effect of screening on long term outcomes, such as lipid concentrations or cardiovascular outcomes in adulthood (Lozano et al., 2016a).

Evidence of clinical outcomes of population genomic screening for FH is emerging, but limited to short-term outcomes. Several studies have found that population genomic screening identified individuals with clinical manifestations of FH who were previously unaware of their condition (Buchanan et al., 2020; Lee et al., 2021b; Elhanan et al., 2022). In these studies, a proportion of individuals with pathogenic FH variants initiated risk-reducing strategies such as LDL-lowering medications (Buchanan et al., 2020; Lee et al., 2021b; Elhanan et al., 2022). In one study in which patients were tasked with informing their healthcare provider of their population genomic screening results, LDL-C levels improved in the short term for only 9% of patients with pathogenic FH-related variants, while the remainder exhibited no change in their clinical management (Elhanan et al., 2022).

## Is there an agreed policy on whom to treat as patients?

There are evidence-based clinical practice guidelines for the management of individuals with pathogenic variants in genes for HBOC, LS, and FH, as described above. It is important to consider that the evidence used to develop these guidelines is largely from cases ascertained through standard diagnostic approaches, as opposed to through population screening-based ascertainment (Murray et al., 2021). Over time, as evidence on penetrance in unselected populations accumulates, management guidelines may need to be updated with specifications for how to manage individuals with disease risk identified through population genomic screening, given the potential reduced penetrance (Murray et al., 2021). This is less likely to be necessary for the genes included in this review than for moderate penetrance genes, given that the penetrance is likely still high in unselected populations and sufficient to warrant clinical intervention.

## Is the test acceptable to the population?

### Views among founder populations

Much of the evidence base for population-based genomic screening is from the three *BRCA1/2* founder variants’ screening in the AJ population. Unselected population-based *BRCA1/2*-screening in the AJ population conducted in Israel (Gabai-Kapara et al., 2014; Lieberman et al., 2017a), Canada (Metcalfe et al., 2013), and the UK (Manchanda et al., 2015a) were found to be safe, acceptable, and feasible. In Israel, Poland, and the UK (Manchanda et al., 2019; Reisel et al., 2022), *BRCA1*/2-screening in the AJ population demonstrates high uptake (> 67%) and satisfaction rates (> 90%), with participants expressing positive attitudes towards the screening experience (Lieberman et al., 2017a). Within the AJ population, motivators for participation were reassurance, decreasing uncertainty, health empowerment, opportunity for risk reduction, and family planning (Lieberman et al., 2017b). Barriers for participation were fear of social and insurance discrimination, stigma, anxiety, and lack of physician awareness and support (Lehmann et al., 2002; Lieberman et al., 2017b). Established founder mutations for LS and FH may also offer a feasible opportunity for population-based genetic screening, however, very limited, if any, research has been done in those populations to determine the acceptability of such programs (Lahtinen et al., 2015; Ponti et al., 2015).

### General public views

Current debate centers around whether the same screening principles and findings for populations with founder mutations can be expanded to all populations (Yurgelun et al., 2015; Foulkes et al., 2016). Outside of the AJ population, there is a paucity of research addressing public views and acceptability of a population-based genetic screening program for HBOC, LS, and FH. For HBOC, surveys of unselected females in the US (Rubinsak et al., 2019) and UK (Meisel et al., 2016) demonstrate high interest (> 82%) and acceptability for population-based *BRCA1*/2 screening. Quantitative and qualitative data from a pilot population genomic screening study predicting ovarian cancer risk demonstrate acceptability, feasibility, reduced cancer worry, and no adverse psychological impact (Gaba et al., 2020; Gaba et al., 2022). Universal genetic and cholesterol screening programs for FH in children demonstrated high uptake within the UK (Wald et al., 2016), and were acceptable to the Australian public (Bowman et al., 2019). Public and patient survey and qualitative interview results from the North America (Graham et al., 1998; Watkins et al., 2011), Europe (Berth et al., 2002), and Australia (Dunlop et al., 2021) indicate support for adult population genomic screening for LS.

Motivators for screening participation include eligibility for increased surveillance and treatment, and the benefits for family members (Ten Haaf et al., 2017). Barriers for screening participation include cost, genetic discrimination, test accuracy, and data confidentiality (Ten Haaf et al., 2017). Genetic discrimination, particularly in the context of insurance, employment, and social relationships (Wauters and Van Hoyweghen, 2016), remains a pervasive deterrent to screening amongst the public, despite the existence of policies to protect sensitive genetic information from misuse worldwide (Joly et al., 2017; Kim et al., 2021).

### Providers’ view

Reported attitudes and views of population genomic screening at the provider level are scarce. Many international studies report that non-genetics specialist healthcare providers (Batra et al., 2002; Carroll et al., 2008; Menzin et al., 2010; Klitzman et al., 2013; Hauser et al., 2018) feel ill-equipped to discuss the benefits, limitations, and health implications of genetic testing for HBOC, LS (Hamilton et al., 2017; Laforest et al., 2019), and FH (Haga et al., 2019; Pang et al., 2020; Watts et al., 2021). Additional reported barriers to population genomic screening include implementation costs, misinterpretation of results, and the potential for increased patient anxiety (Shkedi-Rafid et al., 2013; De Simone et al., 2021). A potential benefit of population genomic screening is the removal of genetic testing eligibility criteria, which providers find overly complex (Klitzman et al., 2013; Laforest et al., 2019).

## Is the cost of case-finding economically balanced in relation to possible expenditure on medical care as a whole?

### Hereditary breast and ovarian cancer

Multiple modeling studies suggest population-based testing for *BRCA1/2* would be more cost-effective than testing based on clinical criteria or family history from a health system perspective in high- and upper-middle income countries (Manchanda et al., 2018; Zhang et al., 2019; Manchanda et al., 2020b; Guzauskas et al., 2020), and cost-saving from a societal perspective (Manchanda et al., 2020b) in high- and upper-middle-income countries. In lower-middle income countries, cost-effectiveness depended on the cost of the test (Manchanda et al., 2020b; Meshkani et al., 2021). Models suggest it may be most cost-effective to initiate population screening among younger individuals (Zhang et al., 2019; Guzauskas et al., 2020). In the AJ population, economic evaluations indicate population genomic screening for *BRCA1/2* variants would be cost-effective (Manchanda et al., 2015b; Manchanda et al., 2017).

### Lynch Syndrome

For LS, economic evidence on population genomic screening among unaffected individuals is limited. A recent U.S.-based economic evaluation suggests that adult population genomic screening among unselected 30-years-old individuals for LS variants would likely be cost-effective at a $150,000 willingness-to-pay threshold (Guzauskas et al., 2022). In contrast, another study found that population genomic screening for LS in unaffected individuals at age 20, followed by cascade testing of first-degree relatives, would not be cost-effective compared to current practices (Dinh et al., 2011). An Australian economic evaluation found that population genomic screening for *MLH1* and *MSH2* for LS would be cost-effective if conducted as part of a multigene panel including *BRCA1/2*, but not if performed in isolation (Zhang et al., 2019).

### Familial hypercholesterolemia

Multiple economic evaluations from the UK, Poland, Spain and Australia have found that population genomic screening for FH would be cost-effective from a healthcare system perspective (Marks et al., 2002; Lázaro et al., 2017; Pelczarska et al., 2018; Marquina et al., 2021), and one Australia-based evaluation suggests it would be cost saving from a societal perspective (Marquina et al., 2021). There is some evidence to suggest that greatest health gains could achieved by screening the youngest probands, however this would also be more costly (Pelczarska et al., 2018). Cascade testing of first- and second-degree relatives of identified patients with FH is also recommended and has been found to be highly cost-effective (Marks et al., 2002; Wonderling et al., 2004; Oliva et al., 2009; Nherera et al., 2011).

## Are facilities for diagnosis and treatment available?

Current models of genetics care are personnel- and time-intensive and not feasible at a population-scale. Key challenges include critical workforce shortages, which contribute to long wait times, a lack of genetics education among non-genetics specialist healthcare providers, and fragmentation of care (Suther and Kiros, 2009; Hann et al., 2017; Office of the Auditor General, 2017; Hoskovec et al., 2018; Stoll et al., 2018; Dragojlovic et al., 2020). These challenges persist in urban areas and are exacerbated in remote and under-served communities (Office of the Auditor General, 2017). Capacity to sustain population genomic screening must also include laboratory infrastructure, secure data storage, as well as bioinformatic and analytic pipelines to support population-scale genomic analyses (Kelly et al., 2021). There is a paucity of data on the availability and distribution of laboratory infrastructure and personnel including clinical laboratory geneticists and medical laboratory technicians (Dragojlovic et al., 2020). This is critical to understand as it will be variable across jurisdictions and will be important for decision-makers to determine how to deliver the program (i.e. the distribution of testing centres).

## Is case finding a continuing process?

The possibility for variants to be reclassified over time means that case finding must be an ongoing process. Most reclassifications are from VUS to likely benign or benign, and reclassification of variants initially classified as pathogenic/likely pathogenic is very rare (Macklin et al., 2018; Mersch et al., 2018; Mighton et al., 2019). In the context of population genomic screening, reclassifications from VUS to pathogenic/likely pathogenic are particularly relevant, as an upgrade from VUS to pathogenic/likely pathogenic could impact medical management. This raises questions about the need for periodic reanalysis and recontact of patients for the return of updated results. The issues of reclassification and recontact already present practical and resource challenges in the context of targeted, clinical testing (Otten et al., 2015; El Mecky et al., 2019), and would be magnified if testing were implemented at the population scale. This is critical to note as non-European populations consistently have higher VUS rates due to lack of representation in databases, leading to higher rates of reclassification and the need for recontact in these populations (Popejoy and Fullerton, 2016; Slavin et al., 2019; Buchanan et al., 2020; Popejoy et al., 2020). There are currently variation in recontact guidelines and practices across jurisdictions, laboratories, and health systems (Bombard and Mighton, 2018; Sirchia et al., 2018), despite recontact being expected by patients (Linderman et al., 2016; Mighton et al., 2021b).

## Ethical considerations

It is important to consider the potential harms and unintended consequences of population genomic screening. Early detection and preventive strategies for HBOC, LS, and FH such as high intensity surveillance, prophylactic surgeries, and pharmacotherapy are not without risks including exposure to radiation, false positives, surgical complications, and adverse drug reactions.

For HBOC, there is some observational evidence to suggest that exposure to diagnostic radiation, including mammography, at a young age is associated with increased risk for breast cancer among females with disease-causing *BRCA1/2* variants (Pijpe et al., 2012). A systematic review of the harms of breast cancer screening among average-risk females found that harms included overdiagnosis (at rates of 11%–22% from randomized controlled trials [RCTs]) and false positive results which were associated with elevated anxiety, distress, and breast-cancer specific worry; however, the review only included females at average-risk and excluded those with pathogenic *BRCA1/2* variants (Nelson et al., 2016). Psychological harms have been identified among *BRCA1/2* carriers, related to false positives and living at risk for disease (Metcalfe et al., 2020). With respect to LS, a systematic review of colorectal cancer screening among average-risk individuals found serious adverse events from colonoscopy including perforations and major bleeds, but these events were uncommon in average-risk populations (Lin et al., 2021). However, high-risk patients such as those with LS were excluded from the review (Lin et al., 2021). For FH, the safety profile differs across pharmacologic therapies. For statins and PCSK9 inhibitors, RCTs have found that treatment-related adverse events did not significantly differ between therapy and placebo (Kastelein et al., 2015; Lozano et al., 2016b), though for statins there are sporadic reports of systemic, immunologic, and pain-related adverse events (Lozano et al., 2016b). Bile acid sequestrants have been commonly associated with adverse GI symptoms, and poor palatability (Lozano et al., 2016b).

Across all conditions, potential harms include genetic discrimination which can arise in a variety of settings. This includes insurance discrimination, which is especially relevant in countries such as the U.S. where much of the population must purchase private health insurances (Ridic et al., 2012; Maynard, 2013). Harms may also be caused when carriers face challenges in accessing risk-reducing strategies in jurisdictions without universal healthcare coverage or among historically underserved populations (e.g., rural populations) (Nguyen-Pham et al., 2014; Chandak et al., 2019; Villegas and Haga, 2019). This raises the question of whether it is ethical to offer population genomic screening in the absence of universal coverage of downstream risk-reducing management. Patient harm may also arise if patients who receive negative results from screening are falsely reassured and forego recommended scheduled screening for average risk populations (i.e., age- and family history-recommended screening) although recent evidence suggests this risk may be minimal (Burnell et al., 2022). Conversely, false positive results may lead to overdiagnosis and overtreatment, where patients and family members may undergo unnecessary investigations and potentially life-altering procedures such as prophylactic surgeries. Although these issues also affect patients undergoing family-history based testing, the higher rates of false positive results associated with population screening coupled with a larger number of patients undergoing genetic testing translates to a larger volume of patients who may receive inappropriate and unnecessary medical care.

At present, the balance of benefits and harms of population genomic screening are not well-characterized. This calls into question whether and to what degree the balance of benefits and harms of screening and subsequent interventions for HBOC, LS and FH should be discussed with patients to ensure informed decision-making. Likewise, it remains unclear how to meaningfully obtain informed consent at the population level given the diversity of literacy, health literacy, socioeconomic status, geography, and culture among screened populations. Genomic screening might not be desirable for all people based on their values and preferences, further highlighting the importance of informed decision-making.

Return of results at the population level presents a further issue. Genomic information is uncertain and complex; delivering this information may lead to adverse psychological outcomes (Mighton et al., 2021c). Among patients receiving positive results after genetic testing, there is evidence of increased risk of anxiety, distress and depression (Rew et al., 2010; Wade et al., 2010; Wade, 2019). Certain populations may face additional risks, such as children feeling a loss of autonomy and women who feel burdened with the responsibility of sharing results with relatives (Gaff et al., 2007; Wade et al., 2010; Wade, 2019). Moreover, parents become overprotective of genetically at-risk children and recognize a disruption of the parent-child relationship (Rew et al., 2010). Although these harms are typically rare and transient, genomic screening at a population level will result in a large number of individuals with psychological harms. In addition to high-quality genetic counseling support, there will also be a need for mental health professionals to support these patients and their families. Furthermore, how to manage VUS in population screening remains unresolved, though there is growing consensus that VUS should not be reported in screening contexts (Murray et al., 2021; Burke et al., 192022). An alternative approach is to examine strategies for return of VUS findings, reclassification, and follow-up, a focus of current investigation.

## Equity

Equity is an important consideration. There are currently disparities in access to and outcomes of genetics services. Racialized and underserved populations often have lower referral rates, differential rates of service uptake, more frequent misdiagnoses or inconclusive test results, older age and more advanced disease stage of diagnosis, and higher mortality rates (Armstrong et al., 2005; Maddison et al., 2011; Cragun et al., 2015; Kerner et al., 2015; Purificacion et al., 2015; Manrai et al., 2016; Vohnout et al., 2016; Amrock et al., 2017; Landry and Rehm, 2018; Muller et al., 2018; Hendricks-Sturrup and Lu, 2019; Ndugga-Kabuye and Issaka, 2019; Ehrenberg et al., 2021). These disparities are present worldwide, highlighting the pervasiveness of health inequities and an urgency for strategies to address them prior to adoption of population screening, to avoid exacerbating these issues. In addition, many underserved populations have limited guidelines on risk factors or treatment recommendations, making it difficult for clinicians to provide appropriate and effective care (Hann et al., 2017). For example, there is a scarcity of guidelines for breast cancer screening in transgender individuals undergoing gender-affirming hormone therapy (Berro et al., 2020; Rolle et al., 2021).

Furthermore, availability of risk-reducing strategies is not consistent across jurisdictions. For example, the extent (if any) of reimbursement for these interventions will vary by healthcare systems, leading to out-of-pocket costs for high-risk individuals, likely exacerbating existing inequities for underserved populations and undermining the effectiveness of the screening program.

## Gaps, future research, and key implications for practice and policy

### Clinical effectiveness

There is considerable evidence that population genomic screening improves detection of individuals with pathogenic variants for HBOC, LS, and FH compared to family history or clinical criteria-based approaches, identifying individuals who would otherwise be missed. However, with the exception of *BRCA1/2* screening in the AJ population, evidence on whether the improved detection rate translates into improved health outcomes (morbidity, mortality) is lacking (Table 2: Summary of key points and gaps). While there are guideline-endorsed, evidence-based strategies to reduce morbidity and mortality for these conditions, several studies suggest that only a proportion of individuals with pathogenic variants identified through population genomic screening approaches actually uptake the associated risk-reducing interventions (Elhanan et al., 2022). Furthermore, studies on clinical effectiveness and ongoing pilot studies (Foss et al., 2022) have primarily employed observational or retrospective designs which suffer from multiple sources of bias (e.g., missing data, loss to follow up) that could reduce the quality of the evidence. However, among the AJ population, there is substantial evidence to support population screening for *BRCA1/2,* including high acceptability, satisfaction, uptake of preventive strategies, in addition to improvements in long term outcomes and reduced costs (Metcalfe et al., 2010a; Metcalfe et al., 2010b; Metcalfe et al., 2012; Metcalfe et al., 2013; Gabai-Kapara et al., 2014; Manchanda et al., 2015a; Manchanda et al., 2015b; Manchanda et al., 2016; Lieberman et al., 2017a; Lieberman et al., 2017b; Manchanda et al., 2017; Manchanda et al., 2019; Manchanda et al., 2020a; Manchanda et al., 2020c; Reisel et al., 2022). Another gap in the literature is that some data has been generated from biobanks and return of secondary findings, which is not reflective of population genomic screening and its outcomes. There is a need for large-scale, prospective, purpose-built population genomic screening pilot studies designed to capture long-term outcomes (Table 3: Recommendations/future directions). While RCTs provide a higher level of evidence than observational studies (Brozek et al., 2009), it may not be warranted to screen only half the population given a lack of equipoise. However, RCTs could be conducted where appropriate, such as for refining the strategy of undertaking testing (e.g., comparing different models for obtaining consent or returning results).

**TABLE 2:** Summary of key points and gaps.

	*BRCA1/2*-associated HBOC	LS	FH
Natural history	Caused by pathogenic variants in *BRCA1/2.* Other genes cause breast and ovarian cancer, however, they are out of scope of this manuscript.	Caused by pathogenic variants in the mismatch repair genes *MLH1, MSH2, MSH6, PMS2*, as well as deletions in *EPCAM*.	Caused primarily by pathogenic variants in *LDLR*, *PCSK9*, and *APOB*.
	High penetrance for female breast, and ovarian cancers among others.	High penetrance for CRC, endometrial cancer, and ovarian cancer among others.	Elevated low-density lipoprotein cholesterol (LDL-C), which leads to risks for cardiovascular disease and premature mortality.
	All conditions: Underdiagnosed in the general population. Limited evidence on penetrance in the general population.		
Test and intervention characteristics	NGS is effective, and could be coupled with gene-targeted deletion/duplication analysis to increase detection of pathogenic variants.	NGS is effective, and could be coupled with gene-targeted deletion/duplication analysis to increase detection of pathogenic variants. *PMS2* testing should be carried out by experienced laboratories as homologous pseudogenes present challenges and variants require validation. Deletion/duplication analysis is required for detecting disease-causing *EPCAM* variants.	NGS is effective, and could be coupled with deletion/duplication analysis to increase detection of pathogenic variants.
	There are guideline-endorsed, effective options for risk-reduction: prophylactic bilateral mastectomy, prophylactic bilateral salpingo-oophorectomy, surveillance with MRI and mammography, chemoprevention.	There are guideline-endorsed, effective options for risk-reduction: surveillance colonoscopy, prophylatic, hysterectomy, prophylactic, bilateral salpingo-oophorectomy, chemoprevention with aspirin.	There are guideline-endorsed, effective options for risk-reduction; Guideline-endorsed, effective options for risk reduction: pharmacologic treatments which are effective at reducing LDL-C levels.
Clinical and cost-effectiveness	Population genomic screening increases detection rate vs. family history-based approaches.	Population screening increases detection rate vs. family history-based approaches.	Population genomic screening increases detection rate vs. family history-based approaches.
	Improved short-term outcomes from high-risk screening or prophylactic surgeries, and some long-term psychological outcomes for AJ populations.	One cost-effectiveness analysis suggests that population genomic screening for LS in US context would be cost-effective at $150,000 USD threshold; an Australian analysis suggests population genomic screening for LS genes (*MLH1, MSH2*) alongside HBOC genes would be cost-effective, but would not be cost-effective in isolation.	Modelling studies suggest that universal cholesterol screening followed by genomic testing and cascade testing of relatives would be cost-effective from a health system perspective and cost saving from a societal perspective.
	Economic models suggest population screening would be cost-effective in the general population compared to family history/clinical criteria-based screening in high- and middle-income countries, and cost-effective or cost-saving in the AJ population.		
Next steps/needs in order to advance population screening	There is evidence to support population genomic screening in the AJ population.	There is some limited evidence that population genomic screening for LS leads to uptake of risk reducing strategies, however more evidence on clinical outcomes is needed, as well as cost-effectiveness models from jurisdictions other than the US.	There is some evidence that population genomic screening for FH improves detection rate and short-term outcomes, and cost-effectiveness models suggest it would be cost-effective, however evidence on long-term health outcomes of population screening (cardiovascular events, mortality) is needed.
	Pilot implementation studies in the general population have been initiated in Australia and the UK; (Lacaze et al., 2022; Yorkshire Cancer Research, 2022) more are needed in other jurisdictions.		
	All conditions: Prior to implementing population screening, public engagement with rigorous, evidence-based approaches is needed, and economic evaluations should be conducted in context of the healthcare system in which screening implementation is being considered. Population genomic screening will require major investments in infrastructure and workforce capacity-building; decision makers will need to determine how population genomic screening should be prioritized relative to other healthcare programs.		

**TABLE 3:** Recommendations and future directions.

Recommendation	Considerations
Future programs and research should consider equity	Consider equity at all points in the care pathway
	Representation of the diverse voices within the population is crucial to inform the design and implementation of a population screening program and necessary for the full potential of genomic screening to be realized
Long term, high quality, studies of clinical effectiveness are needed	To date, most studies have reported on short-term, surrogate outcomes. Longer term studies that assess morbidity and mortality are needed
Cost-effectiveness is context-specific; economic evaluations should be conducted from the perspective of the health care system considering implementing screening	Most economic evaluations of genomic technologies have employed modeling or been conducted within the AJ population. Real-world data in other populations is needed
	Pilot population genomic screening programs and research studies should include concurrent cost-effectiveness analyses
Optimize capacity/workforce	There are critical shortages in the genetics workforce and laboratory infrastructure
	Scaling up the genetics workforce, capacity-building for non-genetics healthcare providers will be needed to support population screening
	Use of digital tools and automation can promote efficiency and enable capacity for population screening
Large-scale studies are needed to characterize penetrance of Tier 1 conditions in unselected populations	The cohorts under study should include individuals of diverse ancestries
	Future work is needed to assess the contributions of polygenic, monogenic, and other risk factors to disease risk in order to improve risk prediction
	Risk prediction should incorporate complex modeling (e.g., BOADICEA) to incorporate multiple risk factors
Implement population-based *BRCA1*/*2* testing in the AJ population	There is sufficient evidence to support population screening for *BRCA1/2* in the AJ population
	Pilot implementation studies in the (non-AJ) general population are needed

### Acceptability

Successful implementation of population genomic screening depends on its acceptability to both the participants and providers, as it can reveal critical issues that can impact uptake, and program compliance (Screening programmes: A short guide, 2020). Much of the current evidence remains within the context of the AJ population for HBOC, which limits the transferability of these findings to the general population and for LS and FH contexts. Rigorous, evidence-based approaches to engage with the public and providers can include public deliberation (Siegel et al., 2013), discrete choice experiments (DCE) (Reed Johnson et al., 2013; Miller et al., 2015; Hauber et al., 2016; Marshall et al., 2016; Terris-Prestholt et al., 2019; Mighton et al., 2021b), or interviews and focus groups (Abelson et al., 2003). Diverse views on expectations and acceptance for the entire trajectory of population genomic screening (e.g., from invitation for screening to follow-up care) within the target jurisdiction, are required to justify the need and to inform the design and implementation of a public health program of this magnitude.

### Economic evaluation

Economic evaluations of population genomic screening have had some limitations. Most have been conducted from the health system payer perspective, which is the perspective which typically informs health system decision-making. However, economic evaluations from a health system perspective do not capture out-of-pocket or indirect costs to patients and family members. More economic evaluations from societal perspectives that capture out-of-pocket and indirect costs borne by patients and family members are needed given the impact of results on relatives and their spill-over effects (Caro et al., 2012; Drummond et al., 2015; Husereau et al., 2022). Important contextual factors to consider include test costs and funding and implementation of healthcare (e.g., single-payer/universal healthcare systems vs. private health insurers). For example, in the US, where a large portion of funding is provided by various private insurers, implementation of a coordinated, public health screening program for the entire country will face challenges. Existing economic evaluations have used modeling to evaluate cost-effectiveness; yet models are limited by their assumptions and model inputs. Real-world evidence on the economic impacts of population genomic screening, is therefore needed. Furthermore, variations in cost-effectiveness thresholds exist between jurisdictions (e.g., $100,000/QALY gained). Decisions about population screening are highly context specific, and decision makers will also need to consider what the greatest public health priorities are in their jurisdiction.

### Programme infrastructure and workforce

In order for population genomic screening to be feasible, there is a need to scale up the genomics workforce, build capacity among non-genetics healthcare providers, and incorporate alternative models of service delivery (Cragun et al., 2015; Peterson et al., 2020) such as mainstreaming (Hamilton et al., 2021; Scheinberg et al., 2021; McCuaig et al., 2021; Yoon et al., 2021; Piedimonte et al., 2020; O'Shea et al., 2021; Ramsey et al., 2022) and the use of digital tools (Manchanda et al., 2016; Bombard and Hayeems, 2020; Shickh et al., 2021; Lee et al., 2022). The use of digital decision support tools is particularly promising. There is increasing evidence that when combined with a brief genetic counseling session, they perform as well, if not better than traditional counseling at improving knowledge, satisfaction, risk perception, and communication between family members, while reducing time spent with HCP and costs (Manchanda et al., 2016; Bombard et al., 2020; Solomon et al., 2020; Bangash et al., 2022; Pande et al., 2022). Although tools have been developed for all three Tier 1 conditions, there are a larger number of tools, at more advanced stages of development and implementation for *BRCA1/2* testing, compared to FH and Lynch syndrome (Manchanda et al., 2016; Bangash et al., 2022; Pande et al., 2022). Moreover, improvements in information technology infrastructure, bioinformatics pipelines, data security and corresponding workforce training would improve the management of population scale genetic data (Khoury et al., 2016; Kelly et al., 2021). It is critical that future research incorporates evaluations of alternative service delivery models, coordination and access of a putative population genomic screening program along with follow up care, both of which have been neglected in evaluation frameworks and the literature, but will inform the ultimate success of the programs (Andermann et al., 2008; Andermann et al., 2010; Pitini et al., 2019).

### Equity

There are currently inequities in access to clinical genetics services, and any additional screening or innovations will only continue to serve populations with access to these services unless deliberate focus is placed on engagement and collaboration (Ford and Airhihenbuwa, 2010a; Ford and Airhihenbuwa, 2010b) across underserved populations. Representation of the diversity within the population is crucial to informing the design and implementation of a population screening program that is centered in the margins. Improvements in transparency, representation, and community collaboration must be prioritized at the outset (Lemke et al., 2010; Caulfield et al., 2014). Designing and implementing an accessible and inclusive population screening program offers opportunities to overcome well-characterized barriers of current genetic service models fueled by structural racism, medical distrust, and a history of eugenics (Fine et al., 2005; Ontario Ministry of Health and Long-Term Care, 2018; Fraiman and Wojcik, 2021). With more diverse participants engaging in genetic research, the diversity of genetic databases can improve, leading to more accurate variant interpretation and higher carrier identification for diverse communities (Landry et al., 2018). Until the benefits of screening are accessible to communities who have been historically underserved and marginalized, the full potential of population genomic screening cannot be realized.

## Limitations

Our review has several limitations. This was not a systematic review, nor was a formal quality appraisal of studies conducted. Moreover, this review was limited to Tier 1 conditions-future research and evidence synthesis will be needed to address other actionable gene-condition pairs (e.g., other genes for hereditary breast and ovarian cancer including *PALB2, RAD51C, RAD51D,* and *BRIP1* (Manchanda et al., 2018); *TTR* for hereditary transthyretin amyloidosis (Soper et al., 2021); endocrine tumour genes (Savatt et al., 2022); arrhythmia syndrome genes (Walsh et al., 2022)) and their suitability for population genomic screening.

## Conclusion

Despite these limitations, our review suggests that there is evidence that population genomic screening for HBOC, LS, and FH would improve detection of individuals with pathogenic variants in the causative genes compared to traditional approaches to case ascertainment. For outcomes beyond detection rate, HBOC has the strongest support for population genomic screening, with evidence demonstrating clinical and cost-effectiveness in the general population; real world implementation studies in the general population are needed. In the AJ population, there is substantial evidence on acceptability, satisfaction, different models of implementation, psychological/quality of life outcomes, uptake of preventive strategies, and cost-effectiveness in support of population *BRCA1/2* screening.

LS and FH both have preliminary evidence supporting population genomic screening, but major gaps remain in the literature. For FH, although there is evidence suggesting population genomic screening programs would have clinical and cost-effectiveness, the evidence on long-term outcomes is limited. Furthermore, the evidence on cost-effectiveness is limited to modelling studies. Real-world studies establishing cost-effectiveness and clinical effectiveness over longer follow-up periods are needed. Economic models suggest population genomic screening for LS may only be cost-effective at a very high cost-effectiveness threshold. Further evidence is critical to establish clinical effectiveness of screening for LS in asymptomatic individuals and cost-effectiveness in lower- and middle-income jurisdictions.

In addition to filling in the evidence gaps, ethical concerns such as potential overdiagnosis, as well as issues related to equity and access to testing and follow-up interventions will need to be considered at the program design stage. Adoption of population genomic screening will require major restructuring and investments to scale up the workforce, build capacity in non-genetics providers, adapt alternative delivery models (mainstreaming, digital tools), optimize IT infrastructure and prioritize an approach that is inclusive of historically underrepresented populations to ensure the full potential of population genomic screening can be realized.
